# Ventricular Tachycardia or Fibrillation Storm in Coronavirus Disease

**DOI:** 10.1155/2022/1157728

**Published:** 2022-08-18

**Authors:** Muhammad H. Khan, Obadah Aqtash, David M. Harris, Alexandru I. Costea, Myron C. Gerson

**Affiliations:** ^1^Department of Internal Medicine, University of Cincinnati, USA; ^2^Department of Cardiovascular Medicine, University of Cincinnati, USA

## Abstract

Ventricular tachycardia (VT) or ventricular fibrillation (VF) storm associated with severe acute respiratory syndrome coronavirus 2 infection is a potentially fatal complication; the correlation of these 2 disorders, however, has not been well studied. This retrospective case series examined outcomes of 2 patients who were admitted for repeated implantable cardioverter-defibrillator shocks with or without syncope and observed to have VT/VF storms with COVID-19. Mechanisms of VT/VF storms in COVID-19 are multifactorial including myocarditis, systemic inflammation, hyperadrenergic state, hemodynamic instability, hypoxia, acidosis, and proarrhythmic drugs. A higher incidence of VT/VF storm is observed in patients with comorbidities and those requiring critical care, with some studies reporting increased mortality. In our cohort, 1 of the 2 patients succumbed to the complications from COVID-19, and the other patient was discharged to home in stable condition. Monitoring of life-threatening arrhythmias in the setting of COVID-19 may need to be adopted to prevent morbidity and mortality.

## 1. Introduction

The coronavirus disease (COVID-19) pandemic caused by the severe acute respiratory syndrome coronavirus 2 (SARS-CoV-2) began in December 2019 and continues to spread globally. The virus primarily causes pulmonary infections; however, major cardiovascular complications are commonly reported in higher risk patients with diabetes, hypertension, and hyperlipidemia [1]. Additionally, a higher reported incidence of ST elevation myocardial infarction in COVID-19 indicates a different pathophysiological mechanism promoting an acute overthrombotic status favoring intracoronary vessel thrombosis and ST elevation myocardial infarction [2]. Myocardial injury from SARS-CoV-2 infection can lead to multiple complications, including cardiomyopathies with heart failure or cardiogenic shock, venous thromboembolism, and increased incidence of cardiac arrhythmias. Our study is aimed at highlighting the importance of recognizing malignant ventricular tachyarrhythmia (VT) or ventricular fibrillation (VF) in the acute or convalescent phase of the infection.

## 2. Materials and Methods

This is a single-center, observational case-series study of ventricular arrhythmia in 2 COVID-19 survivors previously treated with automatic implantable cardioverter-defibrillator (AICD). We retrospectively identified patients between the period of 01/2021 and 03/2020, who were admitted to the cardiovascular ICU with VT storm and a confirmed positive COVID-19 based on reverse transcription polymerase chain reaction testing.

## 3. Case Presentation

### 3.1. Case 1

**Figure 1:**
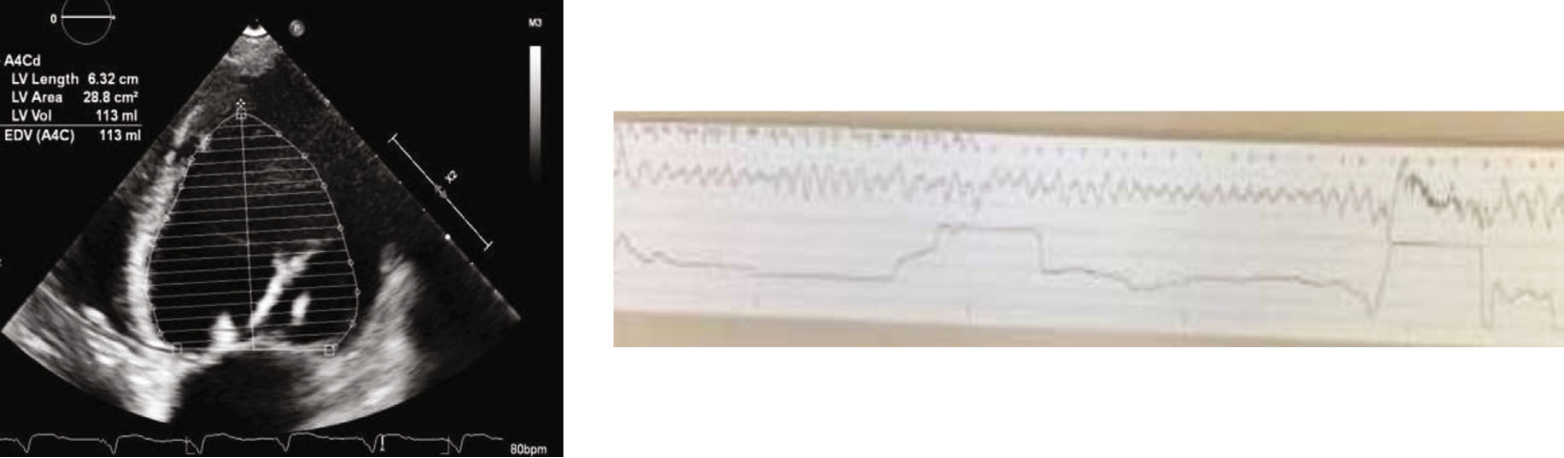
Case 1. Apical four-chamber view of left ventricle with Simpson disk summation method showing increased left ventricular end diastolic volume and diameter. Telemetry strip showing polymorphic ventricular fibrillation rhythm.

A 53-year-old woman presented with a history of repaired ventricular septal defect, atrial fibrillation, tricuspid valve endocarditis with tricuspid valvuloplasty and replacement, coronary artery disease, and biventricular heart failure with reduced left ventricular ejection fraction (40–45%) with biventricular AICD placement. Prior to her hospital visit, she complained of lightheadedness, palpitations, and experienced 1 episode of syncope. She additionally noted that her biventricular ICD had fired approximately 5 times in the past 24 hours which prompted her to present to the hospital for evaluation. Soon after being admitted, she once again lost consciousness and was noted to have VT and VF storm and received 26 shocks. Reverse transcription polymerase chain reaction testing revealed positive SARS-CoV-2. She additionally complained of mild dyspnea and denied any other COVID-19-related symptoms like fevers, chills, diarrhea, headache, or muscle aches. Initial laboratory tests were significant for hypokalemia and low-normal magnesium levels. Transthoracic echocardiography revealed reduced left ventricular ejection fraction (25–30%) with severe diffuse hypokinesis, abnormal left ventricular relaxation, and increased left ventricular filling pressures. Left ventricular size and wall thickness were normal. The bioprosthetic tricuspid valve was functioning normally. She already had coronary angiography performed 4 years ago which was unremarkable other than minor luminal irregularities in the left anterior descending artery. Electrocardiogram on admission illustrated biventricular paced rhythm with a corrected QT interval of 546 ms. AICD interrogation revealed polymorphic VF with 26 appropriate and effective shocks that terminated the arrhythmia (see Figure 1).

Her laboratory data revealed electrolyte imbalance that required replacing potassium and magnesium. She was not on any antiarrhythmic drug at home; however, she used ciprofloxacin (for spontaneous bacterial peritonitis prophylaxis) which was discontinued. She was treated with 150 mg intravenous bolus of amiodarone and then started on infusion at 1 mg/min for 6 hours which resolved her VT. Throughout her hospital course, she was treated with low molecular weight heparin. She briefly required 1-2 liters of oxygen via nasal cannula for dyspnea associated with mild COVID-19 pneumonia, which was successfully treated with a one-time dose of bamlanivimab 700 mg intravenous infusion. Prior to discharge, her QT interval had improved to 385 ms, and she was discharged in stable condition on 200 mg of oral amiodarone daily and 5 mg of Eliquis twice a day.

### 3.2. Case 2

**Figure 2:**
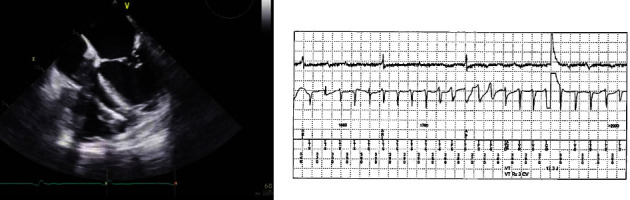
Case 2. Left atrium and moderately dilated left ventricle shown. Pacemaker leads present in the right ventricle. Telemetry strip showing ventricular tachycardia storm.

An 82-year-old man with a medical history significant for coronary artery disease, coronary artery bypass grafting in 1997 complicated by VT with AICD implantation, atrial fibrillation, and atrial flutter with ablation and watchman procedure in 2016 was admitted to the hospital for complaints of exertional angina. He tested positive for SARS-CoV-2, after he underwent RT-PCR testing due to high clinical suspicion. Additionally, he complained of shortness of breath requiring 2-3 liters of supplemental oxygen via nasal cannula. He underwent left heart catheterization which revealed a severe 3 vessel disease with complete occlusion of RCA, no intervention was performed, and a decision was made to pursue medical management given high surgical risk. During his hospitalization, he developed VT storm and received 38 AICD shocks. AICD interrogation revealed 38 episodes of polymorphic ventricular tachycardia, which were not resolved with antitachycardia pacing and were then followed by 38 effective ICD shocks. Transthoracic echocardiogram was performed which revealed a moderately dilated left ventricle with 35–40% systolic function. Severe wall motion abnormality was observed predominantly in the basal to mid inferolateral and basal to mid inferoseptal segments with no evidence of intracardiac thrombus. Pacemaker leads were present in the right atrium and right ventricle (see Figure 2).

During hospitalization, he was treated with multiple intravenous antiarrhythmic agents including lidocaine, amiodarone, and esmolol. He responded well to amiodarone infusion and was subsequently switched to 400 mg twice a day of oral amiodarone with the resolution of VTs on the third day of hospitalization. His AICD settings were adjusted to lower VT detection threshold. His device settings were changed to DDD 60, and the atrioventricular delay was reduced to 180 ms. For afterload reduction, he was treated with Isordil 20 mg every 8 hours and hydralazine 50 mg every 8 hours. Electrocardiogram subsequently revealed an AV dual-paced rhythm at 60 beats per minute. A transesophageal echocardiogram was later obtained which revealed a normal left ventricular systolic function with no signs of valvular vegetations.

Cardiac magnetic resonance and VT ablation were initially considered but later canceled due to worsening of his medical condition with development of septic shock, acute renal failure, and respiratory failure from COVID-19 pneumonia. He required broad-spectrum antibiotics including vancomycin and vasopressors (vasopressin, norepinephrine, and dobutamine) for the treatment of septic shock. Due to worsening of respiratory function, he was intubated, put on mechanical ventilation, and treated with dexamethasone and remdesivir for acute respiratory distress syndrome secondary to COVID-19 pneumonia. He was managed in the cardiovascular intensive care unit during his treatment but eventually succumbed to sepsis and multiorgan failure.

## 4. Discussion

### 4.1. Potential Mechanisms of Ventricular Tachycardia/Ventricular Fibrillation Storm in COVID-19

**Figure 3:**
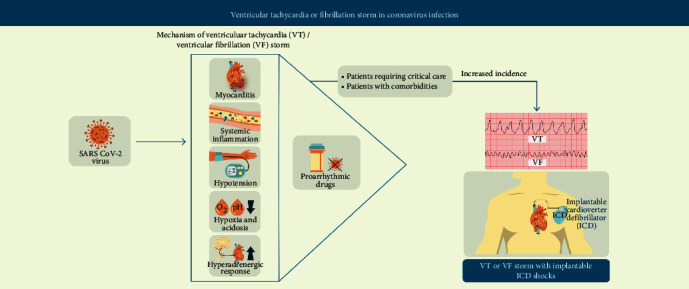
Potential mechanisms of VT/VF storm in COVID-19. Complications resulting from SARS-CoV-2 infection can lead to myocarditis, systemic inflammation, hypotension, hypoxia and acidosis, and a hyperadrenergic response. These along with the use of proarrhythmic drugs can lead to VT/VF storm which are more commonly observed in patients with multiple comorbidities who are critically ill. Abbreviations: COVID-19: coronavirus disease; ICD: implantable cardioverter-defibrillator; SARS-CoV-2: severe acute respiratory syndrome coronavirus 2; VF: ventricular fibrillation; VT: ventricular tachycardia.

SARS-CoV-2 infection predominantly affects the respiratory system but has been causally linked to acute myocardial injury manifesting as elevated cardiac markers, namely, troponin I and T [3, 4]. Several factors can cause myocardial injury following SARS-CoV-2 infection, including type 1 myocardial infarction due to plaque rupture, type 2 myocardial infarction due to increased oxygen demand or reduced supply, myocarditis due to direct viral cytopathic effects, or indirect effects of systemic infection or cytokine storm in a hyperinflammatory state resulting in stress cardiomyopathy [1, 2, 5, 6]. Additionally, patients with hyperglycemia are noted to have a significantly higher levels of inflammatory cytokines that blunt the effects of anti-inflammatory therapy [7]. The predominant mechanisms suspected to be involved in inducing VT/VF storm in the 2 cases reported are myocarditis, systemic inflammation, hemodynamic instability and hyperadrenergic state, hypoxia and acidosis, and use of proarrhythmic drugs. The demographic data and pertinent medical history are shown in Table 1. Mechanisms responsible for the VT/VF storm pertaining to the 2 cases are discussed below (Figure 3).

#### 4.1.1. Myocarditis

Myocarditis is the predominant mechanism of VT/VF storm in this patient cohort, which is marked by elevated cardiac high-sensitivity troponin levels as noted in both the cases described. Myocarditis due to SARS-CoV-2 infection is not generally associated with pathognomonic findings on an electrocardiogram, and often times, the common presenting symptoms of fever, chest pain, and palpitations maybe absent or obscured thus making clinical diagnosis difficult. Two large scale studies have reported acute myocarditis as the cause of SARS-CoV-2-induced myocardial injury. A cardiac autopsy study of 39 confirmed COVID-19 cases proved cardiac infection by documenting the viral genome in myocardial tissue of 60% of patients [8]. Another study identified the SARS-CoV-2 genome in 5 out of 104 endomyocardial biopsy samples with histological assessment revealing myocarditis, necrosis, and granulation tissue [9]. COVID-19-related myocarditis has been documented in several other studies; however, only few studies have demonstrated association between myocarditis and VT/VF storm in the setting of COVID-19-related myocarditis [10–14]. Tachyarrhythmias particularly nonsustained VT have been documented to be related to severity of acute illness and level of troponin elevation and found to be an independent factor of in-hospital mortality [15]. Additionally, SARS-CoV-2-related fulminant myocarditis is now a major consideration in patients who present with chest pain, elevated cardiac troponins and arrhythmias with hemodynamic instability, and left ventricular dysfunction on imaging [16]. These findings suggest early measurement of cardiac biomarkers in COVID-19 may help identify patients at risk of poor clinical outcome as reflected by the mortality of case 2 in our cohort (Table 2) [16].

#### 4.1.2. Systemic Inflammation

Another possible mechanism responsible for ventricular arrhythmia in our patient cohort is cytokine-mediated myocardial dysfunction, which can promote VT/VF storm in severe COVID-19 cases. The presence of inflammatory cytokines, i.e., interleukin-1, interleukin-6, and high-sensitivity C-reactive protein, has been associated with electrical storm in ICD implanted patients in previous studies [17]. Additionally, numerous case studies have documented an association between VT/VF storm and increased inflammatory markers like C-reactive protein in COVID-19 patients, which resolved with the resolution of inflammatory status [18, 19]. The mechanism by which inflammatory cytokines like tumor necrosis factor, interleukin-1, and interleukin-6 propagate arrhythmia involves altering function of cardiac ion channels also known as inflammatory cardiac channelopathy. These cytokines specifically alter the function of gap junctions, outward K^+^ currents, and L-type Ca^2+^ channels in the atrial and ventricular myocytes leading to prolongation of action potential duration and/or QT interval which is associated with malignant arrhythmias [20]. In patients with rheumatoid arthritis, elevated inflammatory markers like IL-6 and CRP are known to be associated with QTc prolongation. Interestingly, both cases in our cohort presented with QTc prolongation > 500 ms and elevated inflammatory markers as shown in Table 2 [21]. This suggests that therapeutic interventions targeting inflammatory cytokines can help in ameliorating the effects of systemic inflammation to reduce acute cardiovascular complications including VT/VF storms while promoting recovery from multiorgan dysfunction.

#### 4.1.3. Hemodynamic Instability and Hyperadrenergic State

Severe SARS-CoV-2 infection can cause sepsis with hemodynamic instability and shock. This can lead to organ level cardiac ischemia resulting in scarring or fibrosis. Functional electrical pathways can form in the scar tissue, creating reentrant circuits that cause ventricular arrhythmias [22]. Although scars are commonly caused by ischemic events, replacement fibrosis can also occur in nonischemic cardiomyopathies. Unfortunately, antiarrhythmic drugs tend to have poor efficacy in VAs due to scars [22].

Alternatively, as a response to cardiac injury, there may be a compensatory response with neurohumoral system activation, leading to sympathetic hyperactivity (hyperadrenergic state) and reduced vagal tone, which helps maintain cardiac output. However, if the cardiac sympathovagal imbalance continues, it creates a maladaptive environment of continued sympathetic activity resulting in cardiac tissue remodeling and ultimately paving pathways for fatal malignant arrhythmias, including electrical storm [23].

#### 4.1.4. Hypoxia and Acidosis

**Figure 4:**
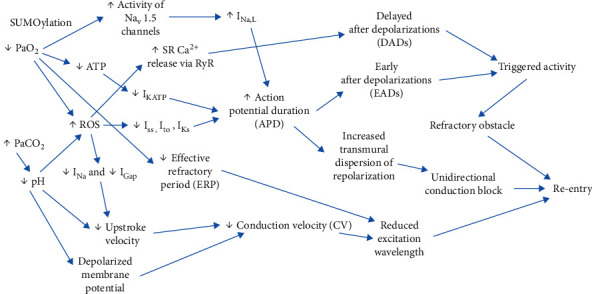
Electrophysiological mechanisms underlying ventricular arrhythmias from hypoxia and hypercapnia [23]. Decreased partial pressure of oxygen, increased partial pressure of carbon dioxide, and decreased pH lead to reduced ATP, reduced upstroke velocity, and increased ROS. These electrophysiological changes with interplay of additional variables promote arrhythmia via non-reentrant and reentrant mechanisms. Source: Lee et al. [25]. Reproduced with permission. Abbreviations: ATP: adenosine triphosphate; PaCO_2_: partial pressure of carbon dioxide; PaO_2_: partial pressure of oxygen; ROS: reactive oxygen species; RyR: ryanodine receptors; SR: sarcoplasmic reticulum.

Multiple studies have reported acute respiratory distress syndrome and pulmonary embolism as common complications of severe COVID-19 [24]. Resultant hypoxia can predispose patients to tachycardia which further increases oxygen demand of the cardiomyocytes. Decreased oxygen in the cardiomyocytes alters the function of multiple ion channels, including the L-type Ca^2+^ ion channel, voltage-gated sodium channels, and outward potassium currents, inducing increased oxidative stress through the generation of reactive oxygen species [25]. Additionally, acidosis secondary to hypercapnia in respiratory failure settings can cause persistent membrane depolarization and reduced phase 0 slope of the cardiac action potential in the ventricular fibers [26]. These changes act as triggers for prolonged action potential, early afterdepolarization, and delayed afterdepolarization [27]. These electrophysiological abnormalities together promote ventricular arrhythmias via both non-reentrant and reentrant mechanisms [27].  Figure 4 summarizes the mechanisms via which hypoxia and hypercapnia can lead to ventricular arrhythmias [25].

#### 4.1.5. Proarrhythmic Drugs

It is crucial to be aware of the proarrhythmic effects of some of the drugs commonly used in the treatment of COVID-19. In the context of systemic inflammation, hemodynamic, and/or metabolic instability, proarrhythmic drugs used in the treatment of COVID-19 should be targeted to reduce the burden of arrhythmias. Case 1 received a one-time dose of bamlanivimab, and case 2 was treated with a 5-day course of dexamethasone and remdesivir in combination. Other than systemic steroids, no other anti-inflammatory drugs were used in the two reported cases.

One group of drugs that has been extensively researched during the COVID-19 pandemic is chloroquine and hydroxychloroquine (HCQ). They are mainly used for their antimalarial properties; however, these drugs received emergency use authorization during the early days of the pandemic based on limited-quality and nonrandomized data. Multiple studies have now concluded that chloroquine or HCQ does not improve outcomes in SARS-CoV-2 infected individuals or act as a prophylaxis to prevent symptomatic infection [28]. Notably, the RECOVERY trial confirmed this analysis by reporting no significant improvement in 28-day mortality with the use of chloroquine or HCQ [29]. Additionally, the chronic use of chloroquine or HCQ is associated with QTc prolongation by binding to and inhibiting the hERG-potassium channel, thereby blocking the delayed rectifier potassium current leading to prolonged ventricular repolarization. Delayed ventricular repolarization enables early afterdepolarizations, which can trigger torsades de pointes and other ventricular arrhythmias [30].

Use of chloroquine or HCQ was initially combined with azithromycin which is used to treat gram-positive and atypical bacterial respiratory infections. Additionally, azithromycin has an anti-inflammatory effect via the downregulation of inflammatory cytokines. Numerous studies at the start of the pandemic reported that patients treated with azithromycin in addition to HCQ had a reduction in detectable viral load, which led to widespread use of the HCQ-azithromycin combination in COVID-19 patients [31]. However, randomized trials have now demonstrated more adverse events, including QTc prolongation in patients who received the HCQ-azithromycin combination [32]. In addition to QTc prolongation, sustained and nonsustained monomorphic ventricular tachycardias have also been reported with the combination of these drugs [33]. Therefore, the CDC recommends against the use of HCQ and azithromycin in hospitalized or nonhospitalized patients as the cardiac risk imposed by the concurrent use of these medications increases the risk of serious cardiac arrhythmias.

Based on anecdotal evidence, protease inhibitors like lopinavir and ritonavir (KALETRA) have also been considered for the treatment of SARS-CoV-2 infection. However, results of 2 large control trials, RECOVERY and SOLIDARITY, have reported failure to reduce mortality, need for mechanical ventilation, or duration of hospitalization [29, 34]. A study on the triple combination therapy of azithromycin, lopinavir/ritonavir, and chloroquine or HCQ resulted in extreme QTC prolongation in 23% of hospitalized COVID-19 patients, with a TdP incidence in 1.14% of study population [35]. Studies have documented association of remdesivir with sinus bradycardia but no such association has been reported with hemodynamic instability or ventricular arrhythmias [36].

### 4.2. Risk and Consequences of COVID-19-Associated Ventricular Tachycardia/Ventricular Fibrillation Storm

Cardiac viral infection with a high inflammatory burden predisposes patients to arrhythmias, especially if other metabolic dysfunctions are present. According to the American College of Cardiology, the overall incidence of arrhythmias due to COVID-19 is 16.7%. The incidence is lower in mild infection (8.9%) and increases to 44.4% in severe illnesses [6]. Supraventricular tachycardia is the most common type of arrhythmia noted in COVID-19 patients [37]. A study reported that sinus tachycardia resulting from hypoxia, hypovolemia, and fever was the most common supraventricular tachycardia, followed by atrial fibrillation [37]. Other supraventricular tachycardias such as atrioventricular nodal reentry tachycardia and conduction blocks continue to be reported but are not well documented due to the benign nature of these arrhythmias [38].

Numerous studies have documented increased proportions of patients developing ventricular arrhythmias in COVID-19. A study by Guo et al. reported VT/VF in 5.9% of COVID-19 patients, with the majority of the cases in patients with myocardial injury [4]. In a large retrospective survey of 827 patients from 12 countries, 21% of the patients admitted for COVID-19 developed ventricular arrhythmias defined as VT, nonsustained VT, or VF. Patients with VT had equal proportions of monomorphic and polymorphic VT (4% each), and 3.4% had VF [39]. Ventricular arrhythmias were associated with significant mortality when compared to atrial arrhythmias, and only 38% of these patients survived to hospital discharge. Other than isolated case reports, observational studies documenting the prevalence of VT/VF storm observed in COVID-19 patients are still lacking [18, 19, 40]. Collectively, these studies suggest that malignant ventricular arrhythmias including VT/VF storm in the context of COVID-19 are more commonly observed in patients with comorbidities and those requiring intensive care unit admission.

### 4.3. Treatment, Prevention, and Monitoring

**Figure 5:**
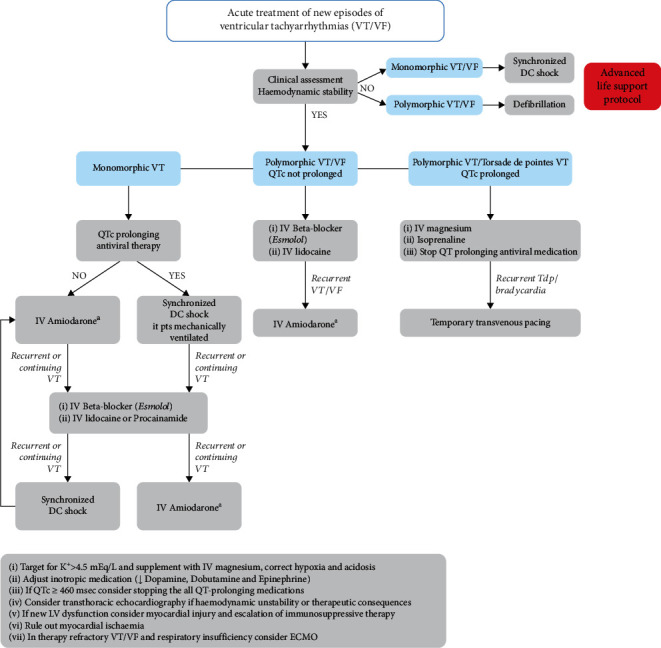
Acute treatment of new episodes of ventricular tachyarrhythmias (VT/VF) [40]. Recommendations for treatment of monomorphic and polymorphic ventricular arrhythmias based on clinical assessment and hemodynamic stability. The benefit of IV amiodarone treatment should be balanced against the polymorphic risk in patients taking QR-prolonging antiviral therapy. Reproduced from https://www.escardio.org/Education/COVID-19-and-Cardiology/ESC-COVID-19-Guidance with permission from The European Society of Cardiology 2021. Abbreviations: DC: direct current; ECMO: extracorporeal membrane oxygenation; IV: intravenous; LV: left ventricle; Pts: patients; TdP: torsade de pointes; VF: ventricular fibrillation; VT: ventricular tachycardia.

COVID-19-related VAs in acute settings should be treated with intravenous amiodarone (American College of Cardiology/American Heart Association–Class of Recommendation 1 and Level of Evidence A). For cases of new malignant VAs unrelated to QT prolongation, imaging via transthoracic echocardiography is warranted to assess ventricular function. Cardiac magnetic resonance imaging should also be considered for myocardial involvement. According to European Society of Cardiology guidelines, intravenous lidocaine is a less effective alternative to amiodarone but may be used, especially if underlying ischemia is suspected (Figure 5) [41]. Temporary pacemaker implantation for overdrive termination or antitachycardia pacing is an option for emergency cases. Following recovery from acute SARS-CoV-2 infection, the need for prevention with either catheter ablation or ICD implantation for secondary prophylaxis should be considered [41]. Additionally, a personalized approach for ICD programming is required to balance unnecessary ICD shocks vs. detecting potential life-threatening VAs.

The general consensus among experts is that patients who developed overt cardiac disease should be closely monitored and reassessed during the acute phase of the SARS-CoV-2 infection. Risk stratifying patients is important during recovery. Some have proposed that patients at high risk should receive repeat transthoracic echocardiography and an electrocardiogram 2–6 months after COVID-19 diagnosis [42]. Additional testing, including Holter monitoring, stress testing, and cardiac magnetic resonance imaging, can be considered in some cases to guide return to normal activity or aerobic exercise, including competitive sports.

## 5. Study Limitations

This study has several limitations. First, given the observational nature of the study, the association between ICD shocks for COVID-19 induced VT/VF storm cannot be interpreted as causal. Second, in patients with a history of arrhythmias, numerous factors other than COVID-19 may play a role in ICD shocks. Finally, this cohort of ICD patients is a unique patient population with large numbers of comorbidities that may not be representative of the general population and hence reduces generalizability of these findings.

## 6. Conclusions

We presented 2 cases of COVID-19-associated VT/VF storm in patients who had multiple ICD shocks delivered. The mechanisms of electrical storm in COVID-19 can be attributed to multiple mechanisms as detailed above. Other than previously published isolated case reports, this is the first case-series study that details the possible mechanisms, risks, and complications of VT/VF storm as a complication of COVID-19. The true burden of electrical storm in COVID-19 may not be fully appreciated due to the incomplete understanding of virus-induced injury to the myocardial conduction system and a large proportion of out-of-hospital mortality. Future investigations of COVID-19-related malignant ventricular arrhythmias are needed to risk-stratify and adopt an effective long-term monitoring and treatment plan in this population.

## Figures and Tables

**Table 1:** Demographic data and relevant comorbidities of patients presenting with ventricular tachycardia.

	Patient 1	Patient 2
Age (years)	53	82
Sex	F	M
Hx of VT	—	+
Hx of AF/AFL	+	+
Hx of CAD	+	+
LVEF (%)	40–45%	35–40%

AF: atrial fibrillation; AFL: atrial flutter; CAD: coronary artery disease; Hx: history; LVEF: left ventricular ejection fraction; VT: ventricular tachycardia.

**Table 2:** Diagnostic test results in patients with COVID-19 and ventricular tachycardia.

	Patient 1	Patient 2
HS troponin (ng/L)	*24*	*3,767*
QTc (ms)	*546*	*596*
BNP (pg/mL)	*232*	*435*
Serum potassium (mmol/L)	**3.1**	3.8
Serum magnesium (mmol/L)	**1.5**	2.5
Venous O_2_ saturation (mmHg)	40	29
Lactic acid (mmol/L)	0.8	2.2
Sepsis	—	+
ARDS	—	+
AKI	+	+
D-dimer (*μ*g/mL)	*6.67*	*>20.0*
Lymphocytes (%)	*11.9*	*10.2*
Ferritin (ng/mL)	*411.5*	223.4
CRP (mg/L)	6	*158.4*

Italics: above normal limit. Bold: below normal limit. AKI: acute kidney injury; ARDS: acute respiratory distress syndrome; BNP: brain natriuretic peptide; CRP: C-reactive protein; HS: high sensitivity.

## References

[1] D’Onofrio N., Scisciola L., Sardu C. (2021). Glycated ACE2 receptor in diabetes: open door for SARS-COV-2 entry in cardiomyocyte. *Cardiovascular Diabetology*.

[2] Marfella R., Paolisso P., Sardu C. (2021). SARS-COV-2 colonizes coronary thrombus and impairs heart microcirculation bed in asymptomatic SARS-CoV-2 positive subjects with acute myocardial infarction. *Critical Care / the Society of Critical Care Medicine*.

[3] Huang C., Wang Y., Li X. (2020). Clinical features of patients infected with 2019 novel coronavirus in Wuhan, China. *In The Lancet*.

[4] Guo T., Fan Y., Chen M. (2020). Cardiovascular implications of fatal outcomes of patients with coronavirus disease 2019 (COVID-19). *JAMA Cardiology*.

[5] de Vries A. A. F. (2020). SARS-CoV-2/COVID-19: a primer for cardiologists. *Netherlands Heart Journal: Monthly Journal of the Netherlands Society of Cardiology and the Netherlands Heart Foundation*.

[6] Imazio M. COVID-19 as a possible cause of myocarditis and pericarditis. American College of Cardiology. https://www.acc.org/latest-in-cardiology/articles/2021/02/05/19/37/covid-19-as-a-possible-cause-of-myocarditis-and-pericarditis.

[7] Marfella R., Paolisso P., Sardu C. (2020). Negative impact of hyperglycaemia on tocilizumab therapy in Covid-19 patients. *Diabetes & Metabolism*.

[8] Lindner D., Fitzek A., Bräuninger H. (2020). Association of cardiac infection with SARS-CoV-2 in confirmed COVID-19 autopsy cases. *JAMA Cardiology*.

[9] Escher F., Pietsch H., Aleshcheva G. (2020). Detection of viral SARS-CoV-2 genomes and histopathological changes in endomyocardial biopsies. *ESC Heart Failure*.

[10] Sala S., Peretto G., Gramegna M. (2020). Acute myocarditis presenting as a reverse Tako-Tsubo syndrome in a patient with SARS-CoV-2 respiratory infection. *European Heart Journal*.

[11] Zeng J.-H., Liu Y.-X., Yuan J. (2020). First case of COVID-19 complicated with fulminant myocarditis: a case report and insights. *Infection*.

[12] Beri A., Kotak K. (2020). Cardiac injury, arrhythmia, and sudden death in a COVID-19 patient. *HeartRhythm Case Reports*.

[13] Hu H., Ma F., Wei X., Fang Y. (2021). Coronavirus fulminant myocarditis treated with glucocorticoid and human immunoglobulin. *European Heart Journal*.

[14] Tavazzi G., Pellegrini C., Maurelli M. (2020). Myocardial localization of coronavirus in COVID-19 cardiogenic shock. *European Journal of Heart Failure*.

[15] Russo V., Di Maio M., Mottola F. F. (2020). Clinical characteristics and prognosis of hospitalized COVID-19 patients with incident sustained tachyarrhythmias: a multicenter observational study. *European Journal of Clinical Investigation*.

[16] Russo V., Bottino R., Carbone A. (2020). COVID-19 and heart: from clinical features to pharmacological implications. *Journal of Clinical Medicine Research*.

[17] Streitner F., Kuschyk J., Veltmann C. (2009). Role of proinflammatory markers and NT-proBNP in patients with an implantable cardioverter-defibrillator and an electrical storm. *Cytokine*.

[18] Mitacchione G., Schiavone M., Gasperetti A., Forleo G. B. (2020). Ventricular tachycardia storm management in a COVID-19 patient: a case report. *European Heart Journal. Case Reports*.

[19] Elsaid O., McCullough P. A., Tecson K. M., Williams R. S., Yoon A. (2020). Ventricular fibrillation storm in coronavirus 2019. *The American Journal of Cardiology*.

[20] Lazzerini P. E., Laghi-Pasini F., Boutjdir M., Capecchi P. L. (2019). Cardioimmunology of arrhythmias: the role of autoimmune and inflammatory cardiac channelopathies. *Nature Reviews. Immunology*.

[21] Lazzerini P. E., Acampa M., Capecchi P. L. (2015). Antiarrhythmic potential of anticytokine therapy in rheumatoid arthritis: tocilizumab reduces corrected QT interval by controlling systemic inflammation. *Arthritis Care & Research*.

[22] Stevenson W. G. (2009). Ventricular scars and ventricular tachycardia. *Transactions of the American Clinical and Climatological Association*.

[23] Kalla M., Herring N., Paterson D. J. (2016). Cardiac sympatho-vagal balance and ventricular arrhythmia. *Autonomic Neuroscience: Basic & Clinical*.

[24] Garcia-Ortega A., Oscullo G., Calvillo P. (2021). Incidence, risk factors, and thrombotic load of pulmonary embolism in patients hospitalized for COVID-19 infection. *The Journal of Infection*.

[25] Lee S., Li G., Liu T., Tse G. (2020). COVID-19: electrophysiological mechanisms underlying sudden cardiac death during exercise with facemasks. *Medical Hypotheses*.

[26] Kagiyama Y., Hill J. L., Gettes L. S. (1982). Interaction of acidosis and increased extracellular potassium on action potential characteristics and conduction in guinea pig ventricular muscle. *Circulation Research*.

[27] Tse G. (2016). Mechanisms of cardiac arrhythmias. *Journal of Arrhythmia*.

[28] Geleris J., Sun Y., Platt J. (2020). Observational study of hydroxychloroquine in hospitalized patients with Covid-19. *The New England Journal of Medicine*.

[29] RECOVERY Collaborative Group (2021). Dexamethasone in hospitalized patients with Covid-19. *The New England Journal of Medicine*.

[30] Jankelson L., Karam G., Becker M. L., Chinitz L. A., Tsai M.-C. (2020). QT prolongation, torsades de pointes, and sudden death with short courses of chloroquine or hydroxychloroquine as used in COVID-19: a systematic review. *Heart Rhythm: The Official Journal of the Heart Rhythm Society*.

[31] Gautret P., Lagier J.-C., Parola P. (2020). Hydroxychloroquine and azithromycin as a treatment of COVID-19: results of an open-label non-randomized clinical trial. *International Journal of Antimicrobial Agents*.

[32] Cavalcanti A. B., Zampieri F. G., Rosa R. G. (2020). Hydroxychloroquine with or without azithromycin in mild-to-moderate Covid-19. *The New England Journal of Medicine*.

[33] Saleh M., Gabriels J., Chang D. (2020). Effect of chloroquine, hydroxychloroquine, and azithromycin on the corrected QT interval in patients with SARS-CoV-2 infection. *Circulation. Arrhythmia and Electrophysiology*.

[34] WHO Solidarity Trial Consortium, Pan H., Peto R. (2021). Repurposed antiviral drugs for Covid-19 - interim WHO solidarity trial results. *The New England Journal of Medicine*.

[35] Russo V., Carbone A., Mottola F. F. (2020). Effect of triple combination therapy with lopinavir-ritonavir, azithromycin, and hydroxychloroquine on QT interval and arrhythmic risk in hospitalized COVID-19 patients. *Frontiers in Pharmacology*.

[36] Attena E., Albani S., Maraolo A. E. (2021). Remdesivir-induced bradycardia in COVID-19: a single center prospective study. *Circulation. Arrhythmia and Electrophysiology*.

[37] Wang Y., Chen L., Wang J. (2020). Electrocardiogram analysis of patients with different types of COVID-19. *Annals of Noninvasive Electrocardiology: The Official Journal of the International Society for Holter and Noninvasive Electrocardiology, Inc*.

[38] Long B., Brady W. J., Bridwell R. E. (2021). Electrocardiographic manifestations of COVID-19. *The American Journal of Emergency Medicine*.

[39] Coromilas E. J., Kochav S., Goldenthal I. (2021). Worldwide survey of COVID-19-associated arrhythmias. *Circulation. Arrhythmia and Electrophysiology*.

[40] Vetta F., Marinaccio L., Vetta G., Marchese D. (2020). Electrical storm in a patient with COVID-19 treated with hydroxychloroquine: a case report. *SAGE Open Medical Case Reports*.

[41] ESC guidance for the diagnosis and management of CV disease during the COVID-19 pandemic https://www.escardio.org/Education/COVID-19-and-Cardiology/ESC-COVID-19-Guidance.

[42] Mitrani R. D., Dabas N., Goldberger J. J. (2020). COVID-19 cardiac injury: implications for long-term surveillance and outcomes in survivors. *Heart Rhythm: The Official Journal of the Heart Rhythm Society*.

